# Characterizing Real-World Implementation of Consumer Wearables for the Detection of Undiagnosed Atrial Fibrillation in Clinical Practice: Targeted Literature Review

**DOI:** 10.2196/47292

**Published:** 2023-11-03

**Authors:** Julie K Simonson, Misty Anderson, Cate Polacek, Erika Klump, Saira N Haque

**Affiliations:** 1 Pfizer Inc New York City, NY United States; 2 Premier Inc Charlotte, NC United States

**Keywords:** arrhythmias, atrial fibrillation, clinical workflow, consumer wearable devices, smartwatches, wearables, remote patient monitoring, virtual care, mobile phone

## Abstract

**Background:**

Atrial fibrillation (AF), the most common cardiac arrhythmia, is often undiagnosed because of lack of awareness and frequent asymptomatic presentation. As AF is associated with increased risk of stroke, early detection is clinically relevant. Several consumer wearable devices (CWDs) have been cleared by the US Food and Drug Administration for irregular heart rhythm detection suggestive of AF. However, recommendations for the use of CWDs for AF detection in clinical practice, especially with regard to pathways for workflows and clinical decisions, remain lacking.

**Objective:**

We conducted a targeted literature review to identify articles on CWDs characterizing the current state of wearable technology for AF detection, identifying approaches to implementing CWDs into the clinical workflow, and characterizing provider and patient perspectives on CWDs for patients at risk of AF.

**Methods:**

PubMed, ClinicalTrials.gov, UpToDate Clinical Reference, and DynaMed were searched for articles in English published between January 2016 and July 2023. The searches used predefined Medical Subject Headings (MeSH) terms, keywords, and search strings. Articles of interest were specifically on CWDs; articles on ambulatory monitoring tools, tools available by prescription, or handheld devices were excluded. Search results were reviewed for relevancy and discussed among the authors for inclusion. A qualitative analysis was conducted and themes relevant to our study objectives were identified.

**Results:**

A total of 31 articles met inclusion criteria: 7 (23%) medical society reports or guidelines, 4 (13%) general reviews, 5 (16%) systematic reviews, 5 (16%) health care provider surveys, 7 (23%) consumer or patient surveys or interviews, and 3 (10%) analytical reports. Despite recognition of CWDs by medical societies, detailed guidelines regarding CWDs for AF detection were limited, as was the availability of clinical tools. A main theme was the lack of pragmatic studies assessing real-world implementation of CWDs for AF detection. Clinicians expressed concerns about data overload; potential for false positives; reimbursement issues; and the need for clinical tools such as care pathways and guidelines, preferably developed or endorsed by professional organizations. Patient-facing challenges included device costs and variability in digital literacy or technology acceptance.

**Conclusions:**

This targeted literature review highlights the lack of a comprehensive body of literature guiding real-world implementation of CWDs for AF detection and provides insights for informing additional research and developing appropriate tools and resources for incorporating these devices into clinical practice. The results should also provide an impetus for the active involvement of medical societies and other health care stakeholders in developing appropriate tools and resources for guiding the real-world use of CWDs for AF detection. These resources should target clinicians, patients, and health care systems with the goal of facilitating clinician or patient engagement and using an evidence-based approach for establishing guidelines or frameworks for administrative workflows and patient care pathways.

## Introduction

### Background

Consumer wearable devices (CWDs) are increasingly being used to monitor fitness and personal health in daily life. Many of these devices have also incorporated medical technology and algorithms to provide alerts for specific physiological changes or abnormalities. There has been early recognition of the potential value of wearable devices in the cardiology setting, especially for cardiac arrhythmias, and key issues have been raised on establishing workflows to process and act on the obtained data [1]. Despite this early recognition, the infrastructure and processes for implementing these devices into clinical practice have not been fully developed.

Atrial fibrillation (AF) is the most common cardiac arrhythmia, affecting an estimated 3 to 6 million individuals in the United States and approximately 46 million people worldwide [2]. Older age has been identified as a primary predisposing factor for AF, and thus, its prevalence is expected to increase owing to the aging of the population [2]. Of particular clinical relevance is that AF is associated with a 4- to 5-fold increase in the risk of stroke [2], which is a leading cause of disability and results in substantial morbidity, mortality, and socioeconomic burden [3,4]. Consequently, the clinical focus on early detection and appropriate management of AF is considered an important component in reducing the subsequent risk and burden of cerebrovascular events, with evidence suggesting that early AF detection and treatment can lead to a 70% reduction in the risk of stroke [5].

Early detection of AF may be especially relevant as it is underrecognized and often undiagnosed because of the frequently asymptomatic nature of this arrhythmia [6,7]; as many as one-third of individuals with AF may be asymptomatic. However, the clinical consequences of asymptomatic AF are likely to be at least similar to those of symptomatic AF, with AF commonly diagnosed based on the occurrence of stroke or other AF sequelae [6]. It has also been reported that asymptomatic AF may be associated with an approximately 3-fold higher risk of cardiovascular and all-cause mortality than symptomatic AF even after adjusting for confounding factors, such as age and CHA2DS2-VASc (congestive heart failure, hypertension, age 75 years and older, diabetes, stroke or transient ischemic attack, vascular disease, age 65 to 74 years, and sex category) score, calculated as congestive heart failure, hypertension, age 75 years and older, diabetes, stroke or transient ischemic attack, vascular disease, age 65 to 74 years, and sex category [8]. Despite the underrecognition of AF and the increased risk of stroke, current evidence on the benefits and harms of AF screening is considered insufficient to determine whether widespread screening should be conducted [9].

Several CWDs have been cleared by the US Food and Drug Administration (FDA) for irregular heart rhythm detection suggestive of AF, including Apple Watch (Apple) [10,11], Galaxy (Samsung) [12], Fitbit (Google) [13], ScanWatch (Withings) [14], and Venu 2 Plus (Garmin) [15]. Although FDA clearance language for CWDs is to detect “irregular heart rhythm suggestive of atrial fibrillation” [10], the use of the term “AF notification” is generally accepted nomenclature in both the medical and lay literature when referring to notifications from CWDs that could be suggestive of AF. Although FDA approval requires rigorous evaluation of the safety and efficacy of pharmaceutical and biological products for high-risk medical devices (class 3), FDA clearance is used for class-2 devices such as CWDs that are considered to be of moderate risk. FDA clearance is granted when the manufacturer has demonstrated that their product is “substantially equivalent to a legally marketed predicate device that does not require premarket approval.” Class-2 devices are also subject to special controls such as specific testing or labeling requirements [16].

The use of mobile devices may be dependent on the type of device and the population [17]. Results from the Apple Heart Study [18] and the Fitbit Heart Study [19] demonstrated that these devices have the ability to identify individuals in the general population who are likely to have AF on subsequent electrocardiogram (ECG) patch monitoring. However, evaluation in patients with known AF has indicated variability in the sensitivity of the Apple device [20,21], which was also supported by a real-world validation study of 5 smart devices that suggested their reduced sensitivity and specificity [22]. Furthermore, the overall value of the widespread use of CWDs such as the Apple Watch for AF detection in the general population has been questioned, with a suggestion that accessing clinical and demographic data from electronic health records (EHRs) could help target these devices to a population that would obtain higher potential benefit of use [23].

Although the value of CWDs for early AF detection to improve outcomes is being further evaluated [24], the potential application of CWDs within the context of AF detection has received limited recognition by medical societies at least in part because of the relatively new nature of this innovative technology and a still developing body of evidence. Although the Heart Rhythm Society (HRS), in a published white paper, provided a meaningful discussion of the benefits and uncertainties of CWDs relevant to specific clinical scenarios encompassing a variety of patient situations [25], they also indicated that more research is needed on how CWDs could best be used. Similarly, a position paper from the European Society of Cardiology (ESC) working group on e-cardiology [26] as well as a collaborative statement from the International Society for Holter and Noninvasive Electrocardiology (ISHNE)/HRS/ European Heart Rhythm Association (EHRA)/and Asia Pacific HRS (APHRS) [27] described the potential role and limitations of these devices in relation to the existing status and operational challenges of mobile health technologies in arrhythmia management. The EHRA also published a practical guide that focused on when and how to use various digital technologies, including CWDs, to detect and manage arrhythmias in different clinical scenarios [28].

The aforementioned papers highlight the need for detailed guidelines on how primary care physicians or cardiologists should use, interpret, or act on information from wearables and are consistent with the key issues that have been previously raised [1]. In a broader sense, these papers underscore that widespread availability of support for real-world implementation of CWDs into clinical and administrative workflows has been lacking as the infrastructure for guiding workflow and subsequent pathways for clinical decisions has not been uniformly established. Thus, greater characterization of these gaps and how they can be filled can facilitate the development of tools for informing patients and clinicians on the use of CWDs and providing guidance to clinicians on data management and appropriate patient follow-up. Such tools could potentially guide the establishment of pathways of administrative workflow among stakeholders, including patients, clinicians, and health care systems.

### Objectives

As a step toward bridging these gaps, our objective was to conduct a targeted literature review to identify articles on CWDs that would enable us to determine the extent of current knowledge on how CWDs can be used for AF detection. Our focus was on three targeted questions: (1) “What is the current state of wearable technology in the use of AF detection?” (2) “What are the operational and technical approaches to implementing wearable technology into clinical workflows?” and (3) “How do healthcare providers and patients view wearable technology for patients with risk of AF?” Rather than reviewing clinical or validation studies on CWDs, these questions were derived with the intent of gleaning information that may be actionable for developing processes and pathways for implementing these devices in clinical practice.

## Methods

### Search Strategy and Selection Criteria

Initial searches were conducted in October 2021 and November 2021 using a combination of Medical Subject Heading (MeSH) terms and keywords (words in the title or abstract), including “remote monitoring,” “telemonitoring,” “wearable device,” “wearable,” “smart watch,” “heart rate,” “arrythmia,” and “atrial fibrillation.” To provide an update, additional searches were conducted in August 2023. All authors contributed to developing search terms under the guidance of JKS and SNH. A full list of the search terms and strings that were used for the searches addressing each of the questions is provided in Tables S1-S4 in Multimedia Appendix 1. The searches were for articles in English only that were published between January 2016 and July 2023. The databases that were searched were MEDLINE via PubMed and the gray literature sources ClinicalTrials.gov, UpToDate Clinical Reference, and DynaMed. For citations considered potentially relevant based on a review of titles and abstracts, the full-text articles were obtained for further review.

Our focus was on articles that applied to real-world implementation of CWDs for AF detection, especially the processes or pathways enabling such implementation. Articles exclusively reporting on or discussing ambulatory monitoring tools, tools available by prescription (eg, Zio Patch monitors), or handheld devices (eg, KardiaMobile) were excluded, as were articles that only tested or reported on detection algorithms or discussed CWDs within the general context of mobile health or digital technology. Other reasons for exclusion of returned citations were the citations being meeting abstracts, validation or clinical studies, study protocols, or deemed out of scope; the reasons for exclusion of full-text articles were insufficient discussion of CWDs or failure to address the targeted questions.

### Collection and Extraction

One author (EK) conducted the searches and reviewed the first-pass results with another author (MA). After this initial assessment, all authors met weekly to review the results and discuss the articles for inclusion or exclusion based on the aforementioned criteria. Subsequent weekly meetings focused on article review and data extraction. The bibliographies of the included articles were further reviewed for any additional potential articles of interest. A qualitative analysis of the included articles was conducted, and we identified emerging themes deemed to be relevant for addressing the 3 targeted questions posed as our study objectives.

## Results

### Search Results

**Figure 1:**
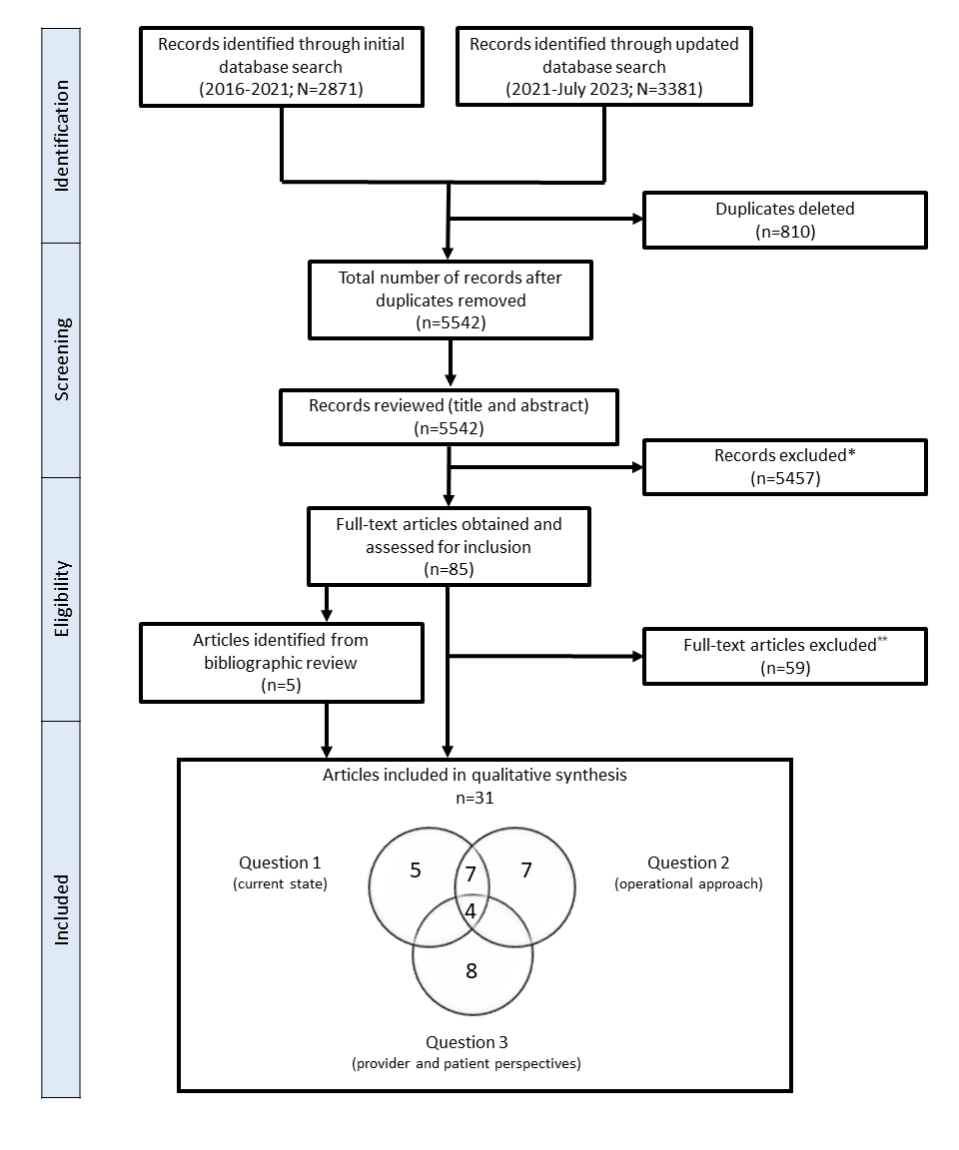
Flow diagram of the included articles. *Reasons for exclusion included the articles being algorithm studies, meeting abstracts, validation or clinical studies, study protocols, or out of scope. **Reasons for exclusion included insufficient discussion of customer wearable devices and failure to address the targeted questions.

On the basis of the search terms in Tables S1-S4 in Multimedia Appendix 1 and as shown in the flow diagram (Figure 1), the initial search strategies returned 2871 citations, and the updated searches returned another 3381 citations (Figure 1). After deletion of 12.96% (810/6252) of duplicates and review of the remaining records, of the 6252 citations, 85 (1.36%) articles were identified for full-text review, of which 26 (31%) were identified for final inclusion, with another 4 identified from bibliographic review (Table 1). Of these 31 articles, 7 (23%) were reports or guidelines from medical societies [27-33], 4 (13%) were general reviews [1,2,34,35], 5 (16%) were systematic reviews [5,36-38], 5 (16%) were health care provider surveys [39-43], 7 (23%) were consumer or patient surveys or interviews [44-48], and 3 (10%) were analytical reports [49-51]. Of these 31 articles, 3 (10%) were from AF-SCREEN, which is a professional organization that advocates for discussion and research on screening for unrecognized or undertreated AF and included a white paper published in 2017 [29], a health care provider survey published in 2020 [39], and a review or report published in 2022 [32].

**Table 1:** Chronological list of the 31 articles identified for final inclusion.

Study, year	Article type	Targeted question addressed^a^
Freedman et al [29], 2017	Medical society report	2
Scott Kruse et al [36], 2018	Systematic review	2
Al-Alusi et al [1], 2019	General review	1 and 2
January et al [30], 2019	Medical society guideline	2
Boriani et al [39], 2020	Provider survey	1, 2, and 3
Chandrasekaran et al [44], 2020	Consumer survey	3
Ding et al [34], 2020	General review	1 and 2
Ding et al [40], 2020	Provider survey	1, 2, and 3
Inui et al [50], 2020	Analytical report	2
Kornej et al [2], 2020	General review	1
MacKinnon and Brittain [35], 2020	General review	1 and 2
Manninger et al [41], 2020	Provider survey	1, 2, and 3
Predel and Steger [49], 2020	Analytical report	1
Hills [46], 2021	Patient survey	3
Hindricks et al [31], 2021	Medical society guideline	2
Lopez Perales et al [17], 2021	Systematic review	1
Manninger et al [42], 2021	Provider survey	1, 2, and 3
Nazarian et al [37], 2021	Systematic review	1
Nuvvula et al [45], 2021	Patient survey	3
Prasitlumkum [5], 2021	Systematic review	1
Smuck et al [51], 2021	Analytical report	2
Varma et al [27], 2021	Medical society report	2
Boriani et al [43], 2022	Provider survey	3
Brandes et al [32], 2022	Medical society report	1 and 2
Ding [52], 2022	Clinical trial with patient survey	3
Faro et al [47], 2022	Patient survey	3
Hermans et al [38], 2022	Systematic review	1 and 2
Leclercq et al [33], 2022	Medical society report	1 and 2
Shih et al [48], 2022	Consumer interviews	3
Svennberg et al [28], 2022	Medical society guidance	1 and 2
Dhingra et al [53], 2023	Cross-sectional population survey	3

^a^Targeted question 1: “What is the current state of wearable technology in the use of AF detection?”; targeted question 2: “What are the operational and technical approaches to implementing wearable technology into clinical workflows?”; targeted question 3: “How do health care providers and patients view wearable technology for patients with risk of AF?”

### Targeted Question 1: What Is the Current State of Wearable Technology in the Use of AF Detection?

#### Technology Options

Detection of AF has traditionally involved the use of a 12-lead ECG or ambulatory ECG monitors such as 24-hour Holter monitoring, and implantable cardioverter defibrillators may be used for long-term monitoring of patients [5,35,37]. However, the value of these methods, especially short-term monitoring for detection, is limited because of the episodic and transient nature of AF as the episodes may not necessarily be captured within the investigation period. The American College of Cardiology, American Heart Association, and HRS guidelines on AF developed before the introduction of CWDs emphasized the potential need for prolonged or frequent monitoring to detect episodes of asymptomatic AF [5,54]. The advent of digital technology has introduced a wide range of mobile devices, including handheld devices, implantable loop recorders, ECG patches, and CWDs, that may be appropriate for use in the cardiology setting under a variety of scenarios [28]. CWDs offer a passive and near-continuous approach to health monitoring that can set individuals on a path toward the recognition of AF and identify those who may have asymptomatic presentation of AF. CWDs also allow patients to play a greater role in disease detection [37], with diagnosis ultimately confirmed by their clinician. This approach may also be considered cost-effective for individuals aged >65 years, with calculated cost-effectiveness ratios substantially below conventionally used thresholds indicative of cost-effectiveness [34].

The ability of CWDs to detect AF relies on photoplethysmography (PPG) sensors or a single-lead ECG sensor (Textbox 1), with some CWDs having both systems. PPG is an optical measurement technique that uses a light source and a photodetector, whereas ECG sensors are based on the detection of electric signals. In contrast to ECG, PPG has the potential advantage of passive, near-continuous monitoring [34]. In devices with both PPG and ECG, a PPG notification can be followed by the individual actively conducting an ECG on the device to characterize the waveform, which can then be shown to and interpreted by a clinician during a follow-up. Therefore, these devices may be especially useful for improving early diagnosis in individuals with asymptomatic or paroxysmal AF with short episodes [2]. A review by Al-Alusi et al [1] in 2019 described the early landscape of wearable monitors in the cardiology setting, including a discussion of key questions and challenges that need to be addressed for their implementation. On the basis of the digital technology landscape, the EHRA practical guide provided a flowchart with suggestions for when and how to screen for AF using wearable devices in different populations and clinical scenarios [28].

**Summary of sensor technology used in consumer wearable devices (CWDs) to detect atrial fibrillation (AF).**

**Electrocardiogram (ECG) sensors (Apple Watch, Fitbit, Galaxy, ScanWatch, and Venu 2 Plus)**

- They measure electrical activity of the heart.
- Single-lead ECG devices may represent a cost-effective method for AF detection.
- Using the ECG sensor requires a person to touch the dial (electrode) on the device using their other hand.

**Photoplethysmographic (PPG) sensors (Apple Watch, Fitbit, and ScanWatch)**

- They use light to measure volume changes in microvasculature to monitor heart rate.
- CWDs may have either 2 or 4 PPG sensors.
- The ability for near-continuous monitoring may be an advantage of PPG sensors relative to ECG sensors.

#### CWD Confidence and Concerns

Reviews of digital health technology mentioned the importance of evaluating and establishing the accuracy of such devices [1,27,28,32-34,38]. In particular, the accuracy of CWDs in detecting arrhythmia consistent with AF is integral for instilling confidence in these devices, and this concern was also expressed in some manner in clinician surveys [39-42]. Although randomized controlled trials (RCTs) represent the gold standard for assessment, the number of RCTs that measured the accuracy of CWDs was limited. However, systematic reviews and meta-analyses of non-RCT studies have reported good accuracy. In one meta-analysis that included 5 observational studies of smartwatches, the sensitivity and specificity were 93% and 94%, respectively, and PPG provided slightly better diagnostic accuracy than single-lead ECG, although there was heterogeneity among the studies [5]. Another review of 18 studies, nearly all of which used PPG, estimated that the sensitivity, specificity, and accuracy of smartwatches for the detection of cardiac arrhythmias were 100%, 95%, and 97%, respectively [37]. These analyses support the high diagnostic accuracy of this technology for the detection of cardiac arrhythmias and can convey an increased level of confidence in their use. Both analyses also emphasized the need for further evaluation of “clinical implications and generalizability,” especially given that the technology is noninvasive [5], and the need “to clearly define the ideal population for the use of these systems, as well as to help form specific guidance on the conduct of device-detected disease” [37]. Other systematic reviews of mobile health technologies for AF detection also emphasized the need to target appropriate populations and evaluate clinical outcomes, especially as, despite their generally favorable accuracy, there is the potential for variability depending on how and in whom the device is used [17,38]. This variability further suggests that the comparative effectiveness of the devices should be established in appropriately designed studies [17].

Because of new and novel use of CWD technology for AF notifications, there are few published studies on incorporating these devices into clinical workflows even though the importance of and need for developing the infrastructure for such workflows was recognized in an early review of this technology [1]. In particular, Nazarian et al [37] highlighted that guidance is lacking on what the clinician is expected to do when an individual receives and reports an AF notification. Associated with this lack of guidance is that, even with the high reported accuracy of CWDs, concerns have been raised regarding the potential for false positive rates for AF, which may lead to anxiety, additional health care costs, and potentially inappropriate treatment [34,49]. This type of notification calls into question how to manage the patient population, and recommendations for such management have included the development of appropriate criteria and tools or the use of existing tools such as the Cohorts for Heart and Aging Research in Genomic Epidemiology model for AF (CHARGE-AF) to further guide detection and diagnosis [34]. Other suggestions have been to prioritize individuals at high risk or use threshold criteria that could include age or other risk factors [34], although the use of age-related criteria may represent its own challenge as an older age group may be less accepting of new technology [34,49]; a recent estimate suggests that, in the United States, only 4.6% of smartwatch users are aged ≥65 years [49].

Several articles indicated that there are limited pragmatic studies measuring real-world applications of CWDs [32,34,37,51]. Furthermore, of the few available studies, many excluded data because of insufficient PPG signal quality or were conducted in settings in which individuals were supervised and provided with instructions, thereby introducing potential bias and reducing real-world generalizability [37]. Consequently, as reported by Ding et al [34], there is a need for additional studies to evaluate the deployment, support, and communication strategies for successful AF detection programs using mobile or digital technologies. The review of consumer-led screening for AF published by AF-SCREEN in 2022 also indicated the need for evaluating clinical outcomes associated with consumer-led screening as these outcomes currently remain unknown [32]. This lack of hard end points in the evaluation of CWDs for AF detection has been previously noted in the ISHNE/HRS/EHRA/APHRS statement [27].

### Targeted Question 2: What Are the Operational and Technical Approaches to Implementing Wearable Technology Into Clinical Workflows?

#### Current Approaches

Established or recommended operational and technical guidelines for incorporating CWD AF notifications into the clinical workflow may facilitate consistent patient follow-up and disease management and concomitantly reduce the administrative burden. A specific need for appropriate infrastructure to accommodate workflow was recognized [1], and the updated American College of Cardiology, American Heart Association, and HRS guidelines for management of patients with AF acknowledged that “a role in screening for silent AF may also exist for remote electrocardiographic acquisition and transmission with a ‘smart’ worn or handheld WiFi-enabled device with remote interpretation” [30]. However, these guidelines do not provide specific recommendations for AF detection or practical considerations guiding the incorporation of notifications from such worn or handheld devices into the clinical workflow. Similarly, the ISHNE/HRS/EHRA/APHRS statement mentioned clinical workflow as a component for incorporating mobile health technologies into clinical care but did not offer any recommendations for establishing infrastructure or processes [27].

In the AF-SCREEN white paper from 2017, a proposed screening framework was considered with the additional suggestion of linking to existing workflows [29]. In total, 2 international surveys by Manninger et al [41,42], one published in 2020 and the other in 2021, yielded more practical insights on how clinicians, including electrophysiology specialists, electrophysiology team leaders, cardiologists, and other clinicians, approach incorporating CWDs into clinical practice. In the earlier survey of 417 clinicians, respondents reported that tracings from CWDs suggestive of AF would likely trigger further diagnostic steps, although these “steps” were unspecified in the survey. In the later survey of 539 clinicians comprising the same specialties as the earlier survey, respondents generally reported that they relied on a 12-lead ECG as the next step. AF tracing notifications from a CWD in a symptomatic individual with AF would more likely result in the initiation of anticoagulation treatment than in an asymptomatic individual (59% vs 21%; *P*<.001), whereas PPG recordings would rarely trigger therapeutic intervention. However, the absence of studies assessing therapeutic consequences “from an incidentally diagnosed AF” has also been noted [38].

Subsequent to the studies by Manninger et al [41,42], the 2022 review by AF-SCREEN provided one of the first proposals for a diagnostic clinical pathway following a mobile health AF notification and expanded on the next steps by emphasizing the need for appropriate follow-up regardless of whether the notification was from an ECG or PPG device [32]. The pathway also provided guidance to clinicians depending on whether AF was confirmed (initiate integrated care) or refuted (reassure the patient). The need for guidance on wearable devices was also recognized by the EHRA [28], which proposed pathways that encompassed several important components such as guiding decisions on when and how to choose an appropriate device stratified by patient risk; digital management of AF; and describing the patient’s digital journey, including assessment, AF confirmation, workup and education, and longer-term management. However, the lack of details on how to operationalize wearable devices into clinical and administrative workflows (eg, integrating wearable data with EHR documentation) warrants additional resource development to guide health care systems and other stakeholders.

In the second survey by Manninger et al [42], respondents additionally recognized the need for integration of CWD data into the clinical workflow as well as a need for reimbursement policies to compensate health care providers for collecting and interpreting data. Respondents generally preferred manual incorporation into the workflow: 63% added descriptions of the recordings to the patient’s record, 53% manually uploaded recordings, and only 15.5% used an external platform to access the data. Despite the caveat regarding the lack of workflow recommendations, most respondents (74%) supported systematic screening for AF using CWDs and would diagnose AF based on single-lead ECG (83%) rather than PPG (27%). In contrast, the 2022 AF-SCREEN report did not discuss specific processes necessary for the implementation of CWDs into clinical and administrative workflows, although it did recognize that consumer-led screening could potentially facilitate the early diagnosis of AF and indicate the need for regulatory pathways [32].

#### Challenges and Barriers

The purpose of the published ISHNE/HRS/EHRA/APHRS statement was to define current mobile health technology and outline important challenges and barriers to its incorporation into the clinical workflow [27]. Not surprisingly given the newness of CWDs for AF detection and the lack of pathways guiding their clinical use, the challenges and barriers identified were similar to those consistently noted in other reviews [1,32,33,38]. One of the main barriers was related to workflow, specifically calling out that implementation of mobile health “will require defined aims and fundamental changes to existing workflows and responsibilities” [27]; the need for redefining workflows rather than leveraging current systems has also been suggested to avoid volume overload [1]. Transparency of information was another challenge that affects all stakeholders but may be of particular importance to consumers using these devices as the process of data transparency and accessibility was considered likely to improve engagement between the consumer and the health care system even in the absence of direct actionability by the consumer [27]. In addition to data transparency, the need for ensuring data security and privacy was considered an important challenge for the implementation of wearable devices from the perspective of all stakeholders, including patients [1,27,28,32,33,38].

A systematic review by Scott Kruse et al [36] identified barriers to the implementation of telemedicine such as leadership buy-in, clinician confidence in effectiveness, educating staff, and teaching consumers how to use the technology and access telemedicine. These factors, especially the issue of confidence in effectiveness (ie, accuracy), were also generally recognized as barriers by clinicians in the clinician surveys published subsequent to the review [39-42]. The review further ranked organizational- and consumer-related barriers based on frequency of report [36]. Interestingly, neither workflows nor implementation models were among the top 5 organizational barriers, which included cost, reimbursement, legal liability, privacy or confidentiality, and security of data. However, although most of the studies included in the review were from the United States, other geographic regions were represented that may not necessarily reflect the perspectives of the US health care system. The top 5 barriers that may impede consumer implementation included age, level of education, computer literacy, internet availability, and unawareness of telemedicine products and services. Individual devices may also be associated with specific challenges owing to differences in methods of data collection and device performance [50]. Many of these challenges were echoed in the review or report of digital technology resulting from an ESC roundtable workshop [33].

#### Overcoming Challenges and Barriers

The same ISHNE/HRS/EHRA/APHRS statement that identified challenges and barriers also considered several operational factors that need to be met to overcome these barriers to the successful implementation of mobile health technologies into clinical care [27]. A key factor, which was considered as yet unresolved, was the transmission of data to the clinician, with concerns regarding both logistics (manner of transmission) and practicality (potential for data overload). Other operational needs involved information sharing owing to the lack of organized infrastructure for receiving and managing data as well as for transmitting data and instructions to consumers. Such issues of information sharing likely require a closer interface with EHRs, including the development of defined pathways for sharing and incorporating data. Resolving issues related to cybersecurity was also considered integral to allaying concerns of health care systems and consumers regarding safety and privacy. Although reimbursement is a ubiquitous issue in new health care technologies that require linking potential cost savings to improved outcomes, it was also noted that responsibilities for reimbursement for mobile health technologies “may extend beyond traditional parties in healthcare and drive novel pathways” [27]. However, consumer costs were not specifically discussed, although the affordability of devices has been raised as a crucial consideration, with a need for discussion among patient advocacy groups, health care systems, and insurers for subsidizing their use to limit disparities in care across vulnerable populations [34]. Appropriate solutions to these challenges should be strategically incorporated into the clinical workflow to address information sharing in a way that minimizes burden on clinicians, maximizes confidence, and ensures transparency across multidisciplinary teams. In the survey by Manninger et al [41], 34% of the respondents indicated that they would want the tracing data to be transmitted to a specialized center, whereas 29% and 18% would transmit data directly to the responsible clinician or to the recommending clinician, respectively; only 9% would transmit data to a third party for interpretation.

In a study by Smuck et al [51], 2 successful digital health intervention programs were assessed, albeit for hypertension and diabetes rather than AF, to identify features that they had in common and that could potentially provide a framework to help guide digital health programs in general. Seven common features were identified: (1) a defined role of the wearables within the disease state, (2) integration into the EHR and incorporation of data into the existing data architecture, (3) technology support for consumers, (4) a personalized approach involving support teams rather than just technology solutions, (5) a user-friendly experience for clinicians, (6) a defined reimbursement model, and (7) physician champions and stakeholder opt-in programs. Although potential solutions were provided for factors 2 to 7, these features and their solutions would need to be further evaluated and developed to more specifically address issues related to AF that may be different from those of diabetes and hypertension.

Despite the screening framework and diagnostic pathway proposed by AF-SCREEN [29,32], neither of those publications discussed processes for specifically incorporating CWDs into health care delivery. Although the EHRA provided additional guidance [28], the lack of detailed processes suggests a remaining need for wider recognition and discussion on the implementation and ongoing use of these devices. The ESC report proposes a collaborative approach among stakeholders, including partnership between technology developers and industry leaders, and lists key factors for implementation as well as steps being taken by professional societies [33]. Nevertheless, the processes for facilitating the implementation of CWDs in cardiology have yet to be fully explicated and formalized, although pathways such as those proposed by EHRA [28] can provide a basis for eliciting consensus among health care stakeholders on the development of additional tools and resources for implementing CWDs in clinical practice.

### Targeted Question 3: How Do Health Care Providers and Patients View Wearable Technology for Patients at Risk of AF?

#### Provider Perspectives

Although we sought studies that surveyed the perceptions and practices of providers in the area of CWDs and AF, the surveys that we found were international in scope and mainly represented electrophysiology specialties [39-43]. Many respondents in these surveys believed that CWDs have a role in the potential diagnosis of AF, and they generally knew, used, and recommended such devices. Although 68% of respondents in one survey recognized that CWDs could assist in diagnosis, similar proportions also expressed concern about data overload (69%) [41], suggesting that efficient workflow management of these devices was needed. Most respondents (62%) in that survey also indicated that they would want clear recommendations, such as those from medical societies, on using CWDs and incorporating them into clinical practice. Similar opinions regarding the need for guidance from professional societies were expressed in the other surveys [39,40,42]. However, these surveys were conducted before the 2021 ISHNE/HRS/EHRA/APHRS collaborative statement, although as previously mentioned, even that statement provided few recommendations on workflow. A more recent survey among members of the ESC indicated that, although only a small proportion of respondents (3.5%) reported a lack of trust in digital devices for use in cardiology, there remains a low awareness of the administrative and regulatory aspects of the use of digital devices as well as a need for care pathways for a referral [43]. This survey also highlighted the concerns of clinicians regarding reimbursement issues, although such issues did not preclude the management of presenting patients.

There appeared to be an overall consensus that the available guidelines are not clear on the best clinical practice after AF notification [27,29,31]. All the surveys supported the need to develop appropriate pathways for managing notifications, and most emphasized the importance of defining the population that would most benefit from CWDs as an approach to monitoring for AF [39-43]. Toward this definition, 74% of respondents in one of the surveys supported the use of CWDs based on age and CHA2DS2-VASc scores, starting at medians of 60 years and a score of 2, respectively [42].

Many of these provider perspectives were also summarized in the ESC review of digital technology [33], and although this review also mentioned several potential solutions, there was little discussion of specific initiatives for operationalizing CWD implementation in the cardiology setting.

#### Consumer Perspectives

Even though the overall use of CWDs is driven by consumers, our search identified few studies characterizing consumer perceptions of these devices for use in AF detection. However, a medical society review or report on digital technology summarized some of the key challenges to consumer uptake of such devices, including issues of digital literacy, data privacy, costs, and uncertainty of steps to take subsequent to a notification [33]. Among the surveys, one national survey not specific to the cardiology setting explored the prevalence of CWD use for health care among adults in the United States and evaluated factors that may be predictive of such use [44]. This survey found that 30% of the 4551 respondents indicated use of CWDs, and of these, 47% reported daily use [44]. Although the low prevalence of use may suggest an untapped potential for these devices in health care, the survey also provided information on the demographic using these devices. Interestingly, there was no statistically significant difference in use between those with and without chronic conditions, but the prevalence of use was greater among those with higher education, technology proficiency, and household income, suggesting a need for digital skill development and financial support. Importantly, from a clinical perspective, 82% of respondents indicated a willingness to share data with their clinicians, but other perceptions and needs regarding these devices were not captured.

In a more relevant population of individuals who self-reported the presence or risk of cardiovascular disease, less than one-quarter reported the use of CWDs, and of those who used such devices, approximately 81% did state a willingness to share wearable data with their physicians even though less than half reported daily use [53]. However, the use of CWDs decreased with age and varied according to other demographic factors, including educational attainment and household income, suggesting demographic disparities in availability and use.

Patient use and perspectives on CWDs were also reported in a survey of a focused population of 424 cancer survivors with or at risk of AF [47]. In that survey, 31.8% of respondents reported owning a commercial wearable device, and 79.7% of patients also endorsed arrhythmias as the most important heart condition for detection by such a device. Furthermore, 89.4% of these patients agreed that it would give them peace of mind to know that a commercial wearable device will detect a heart problem. Peace of mind and a sense of security were also attributes associated with wearing a smartwatch by participants in a clinical trial evaluating the accuracy of a smartwatch-smartphone app dyad for the detection of AF among older stroke survivors [52]. However, these patients indicated that in-person training and support enhanced their experience, suggesting the need for a more patient-centric approach to incorporating these devices into clinical practice. A simpler device interface and longer smartwatch battery life were also reported as desirable goals that would improve usability.

The consumer-driven nature of CWDs for AF detection was discussed in a study consisting of interviews with 19 Apple Watch users [48]. These consumers used the device to take ECG readings that ranged in frequency from several times a week to a few times a year and reported that they liked the technical sophistication of performing a function that would normally occur in a clinical setting but were ambivalent about the potential for false positive results that might prompt an unnecessary clinical visit. Although the authors of the study interpreted the consumer reports to some extent as potentially leading to medicalization of CWDs shaped by marketing, these interviews also highlight the need for development of educational materials on the appropriate use of these devices.

Two studies focused on AF surveyed specific populations, with one study conducted at an academic medical center that stratified survey participants by those diagnosed with AF (n=327) and those at risk of AF (n=895), defined as being aged ≥65 years with a CHA2DS2-VASc score of >2 [45]. The other study was conducted among patients with AF by a patient advocacy organization (N=763) [46]. In the former survey, consumers already diagnosed with AF were more likely to share data with their clinician than those at risk of developing AF, although both groups reported similar ownership and use of such devices. In the latter study, most of the patients (71%) were already using CWDs to “monitor or manage their heart rate or rhythm,” although they also expressed concerns regarding accuracy and lack of interest among their clinicians. Although both of these studies were limited by a potential lack of generalizability because of the specificity of the populations, they consistently indicated the existence of knowledge gaps regarding the use of devices and data sharing subsequent to a notification [45,46], further suggesting the importance of developing specific recommendations and broad educational initiatives targeting consumers.

## Discussion

### Principal Findings

Although our goal was to review and summarize the current knowledge of the processes and pathways for implementing CWDs for AF detection in clinical practice, the availability of articles for inclusion in this targeted review was limited, likely because such use of CWDs is relatively new. Even when the search was updated, few relevant articles were identified, and overall, most of the returned citations indicated that CWDs remain an investigational field with less practical discussion on operationalizing their use for AF detection in real-world practice.

The articles that we reviewed in our qualitative analysis emphasize the concerns and needs for effective incorporation of CWDs for AF detection into clinical practice from the perspectives of clinicians and patients. Economic value was also suggested in several articles that indicated that CWDs are likely to be cost-effective for AF detection when used appropriately in populations at risk [29,31,34,41], which has been further supported by a more recent economic simulation model [55].

Several overarching themes were gleaned from this targeted literature review (Table 2), and many of these themes appear to be concerns regarding the use of digital technology in AF detection and cardiology in general [33,38]. A main theme was that, even though professional societies recognize a potential role for these devices, there remains a lack of guidance on the processes that would facilitate the incorporation of incoming data from CWDs. Furthermore, several of the articles, especially clinician surveys, explicitly requested specific guidance and recommendations by professional organizations on workflow and patient management. However, it should also be noted that the current lack of such guidance may be due to another theme that emerged from this review, that is, the fact that there has been limited pragmatic evaluation of real-world applications and outcomes when CWDs for AF detection are incorporated into the clinical workflow. Thus, additional real-world implementation studies are required to determine the best methods for deploying and supporting strategies for successful AF detection based on CWD-derived data. The lack of organized infrastructure for receiving, managing, and communicating CWD data further indicates the technical considerations that need to be addressed to facilitate the incorporation of such data into the EHR.

Another main theme was the need for the development and dissemination of educational resources directed toward clinicians and consumers. In particular, the lack of tools such as care pathways that can both inform clinician engagement with the patient who received a notification and guide subsequent clinical decision-making was considered an important barrier to the use of CWDs. Overcoming this barrier could also be of benefit in addressing the criticism that consumer use of CWDs for health monitoring may be driven by marketing [48,49,56] and would ideally be accomplished via evidence-based pathways developed in conjunction with professional organizations, such as those proposed by the EHRA [28], which can provide a basis for expansion into more detailed clinical pathways and administrative workflows. An integral component of such pathways would be defining who engages with the patients as well as when and how such engagements occur (eg, virtual vs clinic visits); cardiologists and electrophysiologists reported that the use of virtual visits substantially increased as a result of the COVID-19 pandemic [42]. Indeed, the COVID-19 pandemic resulted in a substantial increase in the use of digital health technology by electrophysiology professionals, who also reported concerns regarding an overall lack of supportive infrastructure, including guidelines on clinical workflow [57].

When engaging with patients, there is also a need for educational materials that clearly explain the benefits and limitations of CWDs and what to do should they receive an AF notification on their device. In addition, patient education should unambiguously explain that an AF notification only means that the CWD has detected an irregular heart rhythm and clinician follow-up is required to determine its clinical relevance. As these notifications may result from other sources, including irregular rhythms other than AF, device artifacts, or sudden changes in movement or body position, AF notifications may be open to misinterpretation by patients. Therefore, from the patient’s perspective, clarity regarding the context and meaning of a notification is important for reducing anxiety and informing patients that they should not only provide the notification to their clinician but should also report the circumstances surrounding the notification (ie, when it occurred and what they were doing). In addition, educating patients to capture a single-lead 30-second ECG tracing that accompanies a potential AF notification and then sharing this with the clinician can help determine the appropriate path forward by distinguishing among potential AF, “noise,” or other types of arrhythmias. Such education and follow-up are also important from the clinician’s perspective as these can enable determination of the most appropriate pathway for patient follow-up. Operationally, decisions will need to be made, but who will make the decisions and based on what criteria still needs to be determined. Educational resources should be both proactive to help manage expectations from CWDs and reactive to facilitate engagement and postnotification follow-up.

The range of clinician perspectives on the utility of CWD notifications (PPG vs ECG vs conventionally used technology) and how to manage patients who receive a notification further emphasizes the importance of clinician education on the meaning of an AF notification and establishing diagnostic pathways that consider the potential for false positives, especially in individuals at low risk. False positives also relate to clinicians’ concerns about the potential for data overload from AF notifications as the notification is only for an irregular heart rate, which can arise from different causes (other cardiac rhythm irregularities and circumstantial events) and may not necessarily be an occurrence of AF. These concerns were further reflected by clinicians’ reported desire for tools such as diagnostic and organizational pathways for data management, which would simplify both the workflow and the decision-making process. These tools would provide guidance on when and in whom further follow-up may be appropriate using strategies such as prioritizing patient populations and stratifying by risk. As the clinical implications of an AF notification with regard to diagnosis, treatment, and outcomes are not yet fully understood, additional clinical trials and real-world studies can expand the body of evidence, potentially informing diagnostic pathways and clinical decisions. It should also be noted that issues regarding reimbursement for receiving, analyzing, and responding to CWD data were raised in an international survey of clinicians [42], and clinician reimbursement was a specific focus in a European survey [39], although these issues did not appear to be a barrier to follow-up and patient management. Nevertheless, given the differences in the US health care system, reimbursement policies may need to be considered as part of the frameworks for incorporating CWDs into patient care pathways.

**Table 2:** Themes gleaned from the targeted literature review on the status of consumer wearable devices for detection of atrial fibrillation.

Theme	Description	Needs for resolution
Guidance or recommendations	Lack of guidance on processes for using CWDs^a^ and determining appropriate patient follow-up subsequent to an AF^b^ notification.	Professional societies should play a larger role in providing guidance or endorsement.
Patient age	Age of patients at risk of AF may be relevant to their knowledge and motivation to use new technologies such as CWDs and the internet, along with clinicians’ views of those qualities in their patients.	Develop patient education resources.
Detection methods	Traditional AF detection methods involve 12-lead electrocardiograms and ambulatory monitoring; however, owing to the episodic nature of AF, devices such as CWDs may be able to uncover more cases of irregular heart rhythm.	Conduct clinical trials and real-world studies comparing effectiveness (sensitivity and specificity) and outcomes between traditional detection methods and CWDs.
False positives (accuracy)	How to manage the potentially high rate of notifications of irregular heart rhythm across the patient population.	Identifying patients at risk may help provide balance between screening the general population and addressing all irregular heart rhythm notifications (which are less likely to be undiagnosed AF for low-risk populations); development of diagnostic pathways; guidance or recommendations from professional organizations on workflow and patient management.
Data overload	Organizations may not be able to address the volume of CWD notifications or have mechanisms to triage notifications and follow-up appropriately.	Initiate appropriate organizational infrastructure to address managing notifications and follow-up.
Lack of care pathways	The availability of clinical tools such as care pathways and detailed guidelines for CWDs is limited.	Develop diagnostic pathways and provide guidance from professional societies.

^a^CWD: consumer wearable device.

^b^AF: atrial fibrillation.

Previous surveys evaluating the clinicians’ perspective were from international studies, and although they included clinicians in the United States, the results were not stratified by country. Therefore, it would be valuable to more specifically survey clinicians in the United States as results from international studies may not necessarily reflect their needs and priorities or issues that may be specific to the US health care system. Furthermore, most participants in the surveys were from electrophysiology specialties. As electrophysiologists are potentially more familiar with and confident regarding the use of wearable technology for irregular heartbeat detection, potentially biasing the survey findings and limiting generalizability, a wider range of specialties should be surveyed, including primary care physicians, who are often the main health care contact for patients.

Similar patient-related issues and challenges regarding technology access and acceptance as well as costs were identified by clinicians and in the 2 patient surveys. However, as the patients surveyed represented specific populations, these surveys were likely confounded by selection bias that reduced generalizability [45,46], suggesting that the perspective of patients on the use of CWDs requires further exploration.

A more comprehensive approach to evaluating the effectiveness of CWDs across the patient population may be necessary to determine the potential for care pathways. Such an evaluation of specificity and sensitivity may be especially important with regard to race and ethnicity as there have been equivocal reports on whether skin tone may contribute to the inaccuracy of optical heart rate sensors (ie, PPG) [58].

There is also likely to be varying digital literacy among those at risk of AF as both digital literacy and technology acceptance may vary according to social determinants of health such as age and geographic regions. The age of patients may be especially relevant to their knowledge and acceptance of new technologies [59], as also supported by the results of the survey by Dhingra et al [53], which reported that, among demographic groups with known worse cardiovascular outcomes, those aged ≥65 years have the lowest use of CWDs. As patient-targeted educational resources are lacking for explaining CWD use, guiding expectations, and understanding the meaning of an AF notification, such resources can be developed. These patient-facing educational materials should focus on AF risk factors, use and capabilities of CWDs, and the meaning of an AF notification to allay potential anxiety regarding the receipt of such a notification. The importance of providing sufficient information on the notification to enable the clinician to determine appropriate next steps should also be emphasized in these materials, as should informing the patient of what may be expected for follow-up.

### Study Limitations

The main limitation of this targeted review, as previously mentioned, is that there are few published studies that have evaluated the various stakeholder perspectives on the use of CWDs and how they may be incorporated into clinical practice. It should also be considered that the character of the published literature is still maturing and currently consists mainly of pilot studies or early explorations of the topic that may vary in focus with few RCTs, further emphasizing the emerging nature of CWDs in the cardiology setting. This limitation increases the complexity of making the comparisons that are needed to gather “themes” representing the current state of the evidence. As this was a targeted review rather than a more formal systematic review, it is also possible that we missed some relevant studies.

### Conclusions

This targeted literature review underscores the current lack of a comprehensive body of literature guiding the real-world implementation of CWDs for potential AF detection. Our results provide insights for informing additional research and developing appropriate tools and resources for incorporating CWDs for AF detection into clinical practice. The identified gaps and challenges can provide a focus for surveys and interviews to elicit additional feedback from clinicians and other stakeholders, such as health care systems that have already incorporated CWDs into clinical pathways. Such surveys and interviews will be useful for confirming and prioritizing the most important issues and, when combined with the information gleaned from the targeted literature search, can inform the development of appropriate tools and educational resources. The results of this review should also provide an impetus for the active involvement of medical societies and other health care stakeholders in developing appropriate tools and resources for guiding the real-world use of CWDs for AF detection. These resources should be tailored by stakeholder, such as clinicians, health care organizations, technical and operational staff, and patients. The goals of the resources provided to stakeholders would include establishing guideline-based frameworks for addressing alerts and recommendations for incorporating alerts into administrative workflows and patient care pathways, as well as facilitating clinician or patient engagement. Efforts to fill these gaps and address the identified needs are ongoing and will be reported in future publications. As the use of CWDs in practice increases and the body of medical literature on CWDs grows, expanding the landscape of these devices, development of frameworks and workflows may be able to rely on a more evidence-based approach for incorporating the use of these devices into clinical practice.

## Appendix

Multimedia Appendix 1 Search strategy for wearables for atrial fibrillation, provider perceptions, patient perspectives, and provider acceptance.

## Abbreviations

AF: atrial fibrillation
APHRS: Asia Pacific Heart Rhythm Society
CHA2DS2-VASc: congestive heart failure, hypertension, age 75 years and older, diabetes, stroke or transient ischemic attack, vascular disease, age 65 to 74 years, and sex category
CHARGE-AF: Cohorts for Heart and Aging Research in Genomic Epidemiology model for atrial fibrillation
CWD: consumer wearable device
ECG: electrocardiogram
EHR: electronic health record
EHRA: European Heart Rhythm Association
ESC: European Society of Cardiology
FDA: Food and Drug Administration
HRS: Heart Rhythm Society
ISHNE: International Society for Holter and Noninvasive Electrocardiology
MeSH: Medical Subject Heading
PPG: photoplethysmography
RCT: randomized controlled trial

## Data Availability

The processes for identifying the articles evaluated in this review are included in this published paper and its supplementary files.
