# Supplementary material for: Characterizing Real-World Implementation of Consumer Wearables for the Detection of Undiagnosed Atrial Fibrillation in Clinical Practice: Targeted Literature Review
Source: JMIR Cardio. 2023 Nov 3;7:e47292. doi: 10.2196/47292 (PMC10656655; doi:10.2196/47292)
Supplement: Multimedia Appendix 1 [file cardio_v7i1e47292_app1.pdf]

Supplementary Table 1. Search strategy for wearables for atrial fibrillation.

#	Search string
1	(Wearable Electronic Devices [mh] OR “activity tracker” OR “fitbit” OR “apple watch” OR “smart watch” OR “remote monitor*” OR “biomedical monitor*”)
2	(EKG [tiab] OR electrocard*[tiab] OR electrocardiography [mh] OR arrythmia [tiab] OR Atrial Fibrillation [tiab] OR Atrial Fibrillation [mh] OR “heart rate”)
3	#1 and #2
4	#3 AND ("systematic review" OR meta-analysis OR "evidence review" OR Cochrane)
5	#4 AND eng [la] AND 2016:2022[dp]

Supplementary Table 2. Search strategy for wearables for provider perceptions.

#	Search string
1	(provider* OR “health professional*” OR nurse OR physician OR clinician* OR health personnel [mh])
2	(perception* OR impression* OR view* OR Surveys and Questionnaires [mh])
3	#1 and #2
4	(Wearable Electronic Devices [mh] OR "activity tracker" OR fitbit OR "apple watch" OR "smart watch" OR "remote monitor*" OR "biomedical monitor*" OR mhealth)
5	#3 and #4
6	#5 AND eng [la] AND 2016:2022[dp]

Supplementary Table 3. Search strategy for wearables for patient perspectives.

#	Search string
1	(patient* OR consumer*)
2	(perception* OR impression* OR view* OR Surveys and Questionnaires [mh])
3	#1 and #2
4	(fibrillation)
5	#3 and #4
6	(Wearable Electronic Devices [mh] OR "activity tracker" OR fitbit OR "apple watch" OR "smart watch" OR "remote monitor*" OR "biomedical monitor*" OR mhealth)
7	#5 and #7
8	#7 AND eng [la] AND 2016:2022[dp]

Supplementary Table 4. Search strategy for wearables for provider acceptance.

#	Search string
1	(Wearable Electronic Devices [mh] OR “activity tracker” OR fitbit OR “apple watch” OR “smart watch” OR “remote monitor*” OR “biomedical monitor*”)
2	(EKG [tiab] OR electrocard*[tiab] OR electrocardiography [mh] OR arrythmia [tiab] OR Atrial Fibrillation [tiab] OR Atrial Fibrillation [mh] OR “heart rate”)
3	#1 and #2
4	#3 AND (uptake OR adoption OR acceptance)
5	#4 AND eng [la] AND 2016:2022[dp]
